# Ghrelin Causes a Decline in GABA Release by Reducing Fatty Acid Oxidation in Cortex

**DOI:** 10.1007/s12035-018-0921-3

**Published:** 2018-02-02

**Authors:** Joan Francesc Mir, Sebastián Zagmutt, Mathieu P Lichtenstein, Judit García-Villoria, Minéia Weber, Ana Gracia, Gemma Fabriàs, Josefina Casas, Miguel López, Núria Casals, Antònia Ribes, Cristina Suñol, Laura Herrero, Dolors Serra

**Affiliations:** 1 Department of Biochemistry and Physiology, Facultat de Farmàcia i Ciències de l’Alimentació and Institut de Biomedicina de la Universitat de Barcelona (IBUB), Universitat de Barcelona, Av. Joan XXIII, 27-30, E-08028 Barcelona, Spain; 2 Centro de Investigación Biomédica en Red de Fisiopatología de la Obesidad y la Nutrición (CIBEROBN), Instituto de Salud Carlos III, E-28029 Madrid, Spain; 3 Institut d’Investigacions Biomèdiques de Barcelona, Centro de Investigación Biomédica en Red de Epidemiología y Salud Pública (CIBERESP), Barcelona, Spain; 4 Sección de Errores Congénitos del Metabolismo – IBC, Servicio de Bioquímica y Genética Molecular, Hospital Clínic, IDIBAPS, Centro de Investigación Biomédica en Red de Enfermedades Raras (CIBERER), Barcelona, Spain; 5 Nutrition and Food Science Area, School of Pharmacy, Universidad del País Vasco/Euskal Herriko Unibersitatea, Leioa, Spain; 6 Research Unit on BioActive Molecules, Department of Biomedicinal Chemistry, Institute of Advanced Chemistry of Catalonia (IQAC)/CSIC, Barcelona, Spain; 7 NeurObesity Group, Department of Physiology, CIMUS, University of Santiago de Compostela-Instituto de Investigación Sanitaria, 15782 Santiago de Compostela, Spain; 8 Departament de Ciències Bàsiques, Facultat de Medicina i Ciències de la Salut, Universitat Internacional de Catalunya (UIC), Barcelona, Spain

**Keywords:** Ghrelin, GABA, Fatty acid oxidation, CPT1A, Cortical neurons

## Abstract

Lipid metabolism, specifically fatty acid oxidation (FAO) mediated by carnitine palmitoyltransferase (CPT) 1A, has been described to be an important actor of ghrelin action in hypothalamus. However, it is not known whether CPT1A and FAO mediate the effect of ghrelin on the cortex. Here, we show that ghrelin produces a differential effect on CPT1 activity and γ-aminobutyric acid (GABA) metabolism in the hypothalamus and cortex of mice. In the hypothalamus, ghrelin enhances CPT1A activity while GABA transaminase (GABAT) activity, a key enzyme in GABA shunt metabolism, is unaltered. However, in cortex CPT1A activity and GABAT activity are reduced after ghrelin treatment. Furthermore, in primary cortical neurons, ghrelin reduces GABA release through a CPT1A reduction. By using CPT1A floxed mice, we have observed that genetic ablation of CPT1A recapitulates the effect of ghrelin on GABA release in cortical neurons, inducing reductions in mitochondrial oxygen consumption, cell content of citrate and α-ketoglutarate, and GABA shunt enzyme activity. Taken together, these observations indicate that ghrelin-induced changes in CPT1A activity modulate mitochondrial function, yielding changes in GABA metabolism. This evidence suggests that the action of ghrelin on GABA release is region specific within the brain, providing a basis for differential effects of ghrelin in the central nervous system.

**Electronic supplementary material:**

The online version of this article (10.1007/s12035-018-0921-3) contains supplementary material, which is available to authorized users.

## Introduction

Acyl-ghrelin (hereafter referred to as ghrelin) is the only peripheral hormone with orexigenic effects described to date [1–3]. It is produced in stomach X/A-like cells where both ghrelin/obestatin preprohormone gene and ghrelin O-acyltransferase are expressed [4, 5]. Ghrelin’s main functions are related to the central control of energy homeostasis and its use. Specifically, ghrelin promotes food intake [6–9], increased body weight and adiposity [10–12], controls glucose homeostasis [13, 14] and growth hormone secretion [15], and enhances motivation for food intake [16–18].

Ghrelin functions in hypothalamus are mediated by growth hormone secretagogue receptor (GHSR) [19]. The molecular mechanisms involved under ghrelin activation of GHSR receptor are not completely understood, but in hypothalamus, AMP-dependent protein kinase (AMPK) has a key role in intracellular signal transduction [20–22]. AMPK activation due to ghrelin promotes acetyl-CoA carboxylase (ACC) inactivation, which reduces malonyl-CoA formation and consequently increases CPT1A activity [23]. CPT1A activation triggers a series of molecular events leading to increased expression of orexigenic agouti-related protein (AgRP) and neuropeptide Y (NPY) [24–27], which results in increased appetite.

Ghrelin has also been involved in GABAergic signaling. It has been reported that ghrelin inhibits firing of postsynaptic pro-opiomelanocortin (POMC)-expressing neurons by increasing presynaptic γ-aminobutyric acid (GABA) release [28]. This increase in GABA output in the orexigenic neurons is produced as a consequence of glutamic acid decarboxylase (GAD) expression [29]. Furthermore, CPT1A as an intermediate in ghrelin signaling in ventromedial hypothalamus has been found to increase the expression of vesicular GABA transporter (VGAT), which is considered the factor that controls GABA quantal size, in CPT1A activity-induced hyperphagic rats [30].

Three pathways control the cytoplasmic GABA content to be released: (1) GAD activity, which is the canonical pathway to generate GABA out of glutamate [31]; (2) GABA shunt, which is composed of two enzymatic reactions catalyzed in mitochondria by succinate semialdehyde dehydrogenase (SSADH) and GABA transaminase (GABAT) [32, 33]; and (3) GABA transport into small synaptic vesicles for its release, via VGAT.

Besides energy balance, other actions of ghrelin related to anxiety, cognition, stress, and sleep have been extensively studied [34–37]. For ghrelin to be involved in such processes, it must reach extra-hypothalamic areas. In fact, GHSR was first described in pituitary gland, hippocampus, ventral tegmental area, raphe nuclei, and hypothalamus [38]. However, its expression can be extended to neocortex, olfactory bulb, basal ganglia, and cerebellum [39]. Moreover, 76% of primary cortical neurons are GHSR-positive cells in in vitro culture [40, 41], which indicates that ghrelin may play an important role in these brain areas. However, little is known about the intracellular mediators involved in ghrelin action in cortical neurons.

In this study, we show that ghrelin’s action on cortical neurons involves CPT1A modulation that differs from that observed in hypothalamic neurons. In cortical neurons, ghrelin inhibits CPT1A activity and fatty acid oxidation (FAO), and reduces the levels of Krebs cycle intermediates such as citrate and α-ketoglutarate, GABA shunt enzymes, and GABA release under depolarization conditions. These data indicate that ghrelin modulates GABA in a region-specific fashion, which may account for the variation in ghrelin actions.

## Materials and Methods

### Animals and Treatments

The mice strains used in this project were C57BL/6J and CPT1A^(loxP/loxP)^ mice obtained from HEPD0727_3_H09 clone from the European Conditional Mouse Mutagenesis (EUCOMM) programme. Once the karyotype had been studied, HEPD0727_3_H09 clone was chosen to be microinjected in blastocysts to obtain chimeric mice. Eventually, from the seven chimera that were obtained, we selected one male with 80% chimerism to obtain offspring. This mouse was crossed with C57BL/6J to obtain CPT1A^(+/frt-loxP)^ mice. These mice were genotyped by analyzing the number of *lacz*-containing sequences by digital droplet PCR (ddPCR), as described below. These potentially conditional CPT1A mice were crossbred with C57BL6/J FLP recombinase-expressing mice to eliminate the *lacz* cassette and obtain CPT1A^(+/loxP)^ mice (Supplemental Fig. 1). CPT1A^(+/loxP)^ mice were crossbred to obtain homozygous CPT1A^(loxP/loxP)^ mice.

In order to analyze the effects of ghrelin on several cortical and hypothalamic parameters, we injected a dose of 10 μg ghrelin (or the equivalent volume of phosphate-buffered solution, i.e. 300 μL) intraperitoneally (IP) at 0 min and another dose at 30 min in mice that had been food-deprived for 2 h after the dark period [42]. All the mice received the same amount of ghrelin, as their body weights were similar (25.74 ± 0.52 g for ghrelin-treated mice and 25.52 ± 061 g for the control group). We monitored eating time and food intake for 1 h after the first injection. One hour after the treatment, mice were sacrificed by cervical dislocation and their tissues collected. All the procedures with mice were approved by the Animal Experimentation Ethics Committee of the University of Barcelona (CEEA-UB). DAAM Permit #8173 and colony management Permit C-0020 were obtained from the Government of Catalonia, according to European Directive 2010/63/EU.

### Cell Cultures and Treatments

Primary cortical neurons were directly obtained from fresh cortex as described by Solà et al. [43]. Cerebral cortices from C57BL/6J and CPT1A^(loxP/loxP)^ mice were harvested from unborn pups on embryonic days from 15 to 17. No isolation with less than 60% viability was used. Then, 8 × 10^5^ living cells per well (in 6-well plates) were seeded. Each litter yielded around 6 to 8 plates, which were precoated with 4 °C overnight incubation with 0.005% poly-l-lysine, prepared from 2× solution (Sigma-Aldrich, ref. P4707). Three hours after seeding, cells were attached and the medium was replaced with Neurobasal Medium (GIBCO, ref. 21103-049) supplemented at 1× with B27 (GIBCO, ref. 17504-044) and GlutaMax (GIBCO, ref. 35050-038), as well as 1% penicillin/streptomycin (100 U/mL final concentration) (GIBCO, ref. 15140122). Two days after culturing the cells, 2 μM cytosine β-d-arabinofuranoside (Sigma-Aldrich, ref. C1768) was added to avoid proliferation of non-neuronal cells to the medium. Every 2 days, the medium had to be changed by removing half of the previous medium and replacing it with fresh medium. Cells were prevented from drying out without any medium on them.

To delete CPT1A in the cortical neuron cells, we used CRE recombinase- and GFP-expressing adenoviral vectors (Ad-CRE-GFP) (Vector Biolabs, ref. 1700) with a titration of 4.22 × 10^9^ pfu/mL and Ad-GFP (3.32 × 10^9^ PFU/mL) as a control. We infected the cells with 100 PFU of Ad-CRE-GFP per cell. The medium was changed the next day. The medium used was the same as in the regular culture. Ghrelin treatment was conducted in both primary cortical neurons and GT1-7. Hypothalamic GT1-7 cells were pretreated for 3 h with 5 mM glucose pyruvate- and glutamine-enriched Dulbecco’s modified Eagle medium (DMEM). Primary cortical neurons were pretreated for 3 h with 5 mM glucose Neurobasal®-A medium (GIBCO, ref. 10888022). Then, cells were treated with 100 nM ghrelin (Sigma-Aldrich, ref. G8903) in pretreatment medium for 30 min. To pharmacologically inhibit CPT1A, we added 40 μg/mL etomoxir in the 30-min ghrelin treatment. To block the tricarboxylic cycle by inhibiting isocitrate dehydrogenase, we added 700 μM 2-hydroxyglutarate.

### Metabolic Extracellular Flux XF Analysis

Cortical neuron cells were cultured in customized Seahorse 24-well plates. Before the measurement, cells were treated for 3 h with 5 mM glucose medium. In the last 30 min, the ghrelin treatment was carried out as previously explained. Then, cells were assayed for 1 h in XF Assay Medium (Seahorse Bioscience) plus 5 mM glucose. During the assay, we injected the following at the final concentrations shown: 2 μg/mL oligomycin, 0.16 μM FCCP, and 2 μM antimycin A (Sigma-Aldrich). Oxygen consumption rate (OCR) was calculated by plotting the oxygen tension of media as a function of time (pmol/min), and data were normalized by the protein concentration measured in each well. The results were quantified as the average of 8–10 wells ± SEM per time point in at least three independent experiments.

### Amino Acid Neurotransmitters Release Assay

We carried out the experiments with cultured primary neurons on the 8th day of in vitro (8 DIV) culture, when they were mature enough. If an infection had to be made with Ad-CRE, the cells were infected on 6 DIV, to obtain maximum expression on 8 DIV. On that day, any pretreatment (5 mM glucose reduction for 3 h) and ghrelin treatment with 100 nM ghrelin was completed just before the amino acid neurotransmitter release assay. Two buffers are needed for this assay: Basal 5 mM KCl Hank’s buffer (K5) and depolarizing 90 mM KCl Hank’s buffer (K90). After treatment, the wells were washed with pre-warmed Hank’s Buffer (K5). Then, 1 mL K5 buffer was added per well and incubated for 10 min at 37 °C, so that neurons could stabilize in the buffer. The buffer was removed and cells were incubated with 1 mL K5 for 2 min and the supernatant was kept in a microcentrifuge tube. Then, cells were incubated with 1 mL K90 for 2 min and the supernatant was kept in a microcentrifuge tube. This process was repeated up to six times, alternating between K5 and K90 solutions and keeping the neurotransmitters released into the Hank’s solution for posterior analysis. As a control of non-vesicular neurotransmitter efflux, incubation for 2 min in 1 mL K90 was carried out without Ca^2+^ and 3 mM EGTA, and the supernatant was kept for analysis. All the samples were stored at − 20 °C until analysis and the cells in the plate were lysed with 0.2 mL NaOH 0.2 N to quantify the protein content for normalization. Amino acid neurotransmitter content was then measured from the samples by HPLC-MS/MS at the Institute of Biomedical Research of Barcelona (IIBB-CSIC).

### Digital Droplet PCR

To check if CPT1A^(+/frt-loxP)^ contained the correct integration cassette and the right number of copies in the genome, we genotyped the allele-specific CPT1A^(+/frt-loxP)^ mice previous to generating CPT1A^(+/loxP)^ mice, using a QX100 Droplet Digital PCR System (BioRad, ref. QX100) that had been adapted for the use of QX200 ddPCR EvaGreen Supermix dsDNA binding dye (BioRad, ref. 186-4035). Genomic DNA was obtained from the tail of CPT1A^(+/frt-loxP)^ mice by proteinase K digestion and phenol-chloroform purification. One microgram of genomic DNA was digested for 1 h with 10 U of the restriction enzyme *EcoR*I. To perform ddPCR, the primers used were LACZfor GCTGGAGTGACGGCAGTTAT and LACZrev TACCCGTAGGTAGTCACGCA, and for the control TERTfor CCTCTGTGTCCGCTAGTTACA and TERTrev TCTTTGTACCTCGAGATGGCA. The amplicon sizes were 137 bp for TERT and 197 bp for LACZ. Due to specific characteristics of ddPCR, a temperature gradient was performed to optimize the annealing of the primers. The annealing temperatures tested were 58.9, 60.1, 61.0, and 61.6 °C. The selected temperature was 60.1 °C. The ddPCR mixture for each gene amplification contained the following: 4.4 ng of DNA sample, primer concentration at 0.5 μM each, and 11 μL of EvaGreen® supermix (BioRad) in a 22-μL final volume. A total of 20 μL of reaction mixture and 70 μL of Droplet Generation Oil were placed in the pertinent well of the DG8 cartridge (BioRad, ref. 186-3006). The bubbles were removed from the system, since they can interfere with emulsification. The cartridge was then placed in the QX100 droplet generator (BioRad, ref. 186-3002) for droplet generation. The droplets were transferred to a 96-well PCR plate. The program for amplification started with 5 min at 95 °C, followed by 39 cycles of 30 s at 95 °C and 1 min at 59 °C; finally, the temperature decreased to 4 °C for 5 min, was increased to 90 °C for 5 min, and the reaction was kept at 25 °C. The analysis by the QX100 Droplet Reader (BioRad, ref. 186-3003) and QuantaSoft Software showed that the *tert*/*lacz* ratio is 2.09, which suggest that only one copy of the construct is present in heterozygous CPT1A^(+/frt-loxP)^ mice for each two copies of *tert* reference gene (Supplemental Fig. 1A and B). All the equipment was available from the Laboratory of Luminescence and Biomolecular Spectroscopy (LLEB), Scientific and Technical Services, Autonomous University of Barcelona (UAB).

### mRNA Expression Analysis

Tissues were excised from six mice from each group, frozen, and stored at − 80 °C. Total RNA was extracted from frozen brain tissue using the RNeasy Lipid Tissue Mini kit (QIAGEN, ref. 25-0500-71), following the manufacturer’s indications with minor modifications. Total RNA was extracted from cultured cells using the Illustra Mini RNAspin kit (GE Lifesciences, ref. 25-0500-71), following the manufacturer’s indications with minor modifications. cDNA was obtained using TaqMan Reverse Transcription Kit (Applied Biosystems, ref. N8080234), from 1 μg total RNA from MBH or 400 μg from cell cultures, because of the varying RNA extraction yields. The manufacturer’s protocol was used with hexamer primers to obtain cDNA from all the mRNA. Quantitative real-time polymerase chain reaction (qRT-PCR) was performed using Power SYBR Green PCR Master Mix adapted for LightCycler 480 (Applied Biosystems, ref. 4367659), according to the manufacturer’s indications in the LightCycler 480 Instrument II (Roche, ref. 05015243001). The primers of the genes that were analyzed are described in Supplemental Table 1.

### Analysis of Protein Levels

Protein expression analysis was obtained from four mice from the control group and 4 mice from the ghrelin-treated group. Frozen hypothalamus and cortex were homogenized in protein extraction buffer (30 mM Hepes, pH 7.4, 150 mM NaCl, 10% glycerol, 0.5% sodium deoxycholate [DOC], 1% Triton X-100 with phosphatase and protease inhibitors). Fifty micrograms of protein were analyzed on 10% SDS-PAGE gels and then transferred onto PVDF membranes (Millipore). The following primary antibodies were used: GAD65/GAD2 (1/1000; Cell Signaling ref. 5843), VGLUT2 (1/1000; Cell Signaling ref. 71555), and β-actin (1/50,000; Sigma-Aldrich). Blots were incubated with the appropriate IgG-HRP-conjugated secondary antibody. Protein bands were visualized using the ECL immunoblotting detection system (GE Healthcare) and developed on an ImageQuant LAS4000 mini Fuji luminescence imagining system. The bands were quantified by densitometry using ImageJ analysis software.

### GABA Transaminase Activity

GABA transaminase Assay Kit (BMRService, ref. E134) was used according to the manufacturer’s indications. This kit is based on the sequential GABA transamination reaction and glutamate oxidation, which couples the reduction of iodonitrotetrazolium (INT) into INT-formazan (*ε* = 18 mM^−1^ cm^−1^ at 492 nm).

### Determination of Tissue Acylcarnitine Content

Tissues for analysis were removed quickly, frozen in liquid nitrogen, and stored at − 80 °C prior to quantification. Acylcarnitines were analyzed using an Acquity UPLC-TOF system (Waters) with a BEH C8 column (1.7 μm particle size, 100 mm × 2.1 mm, Waters). The two mobile phases were 1 mM ammonium formate in methanol (phase A) and 2 mM ammonium formate in H2O (phase B), both phases with 0.05 mM formic acid. The following gradient was programmed: 0 min, 65% A; 10 min, 90% A; 15 min, 99% A; 17 min, 99% A; 20 min, 65% A, and a flow rate of 0.3 mL min^−1^. Quantification was carried out using the extracted ion chromatogram of each compound, with 50-mDa windows. The linear dynamic range was determined by injecting standard mixtures. Positive identification of compounds was based on the accurate mass measurement with an error < 5 ppm and their LC retention time was compared to that of a standard (± 2%).

### Neuronal Tricarboxylic Acid Cycle Intermediates Analysis

Analysis of tricarboxylic acids was carried out by gas chromatography-mass spectrometry (GC-MS) detection, with a method adapted from the literature [44–46]. Experiments were performed on cortical cell cultures (2.5 × 10^5^/well). At the end of the incubations, cells were washed with PBS, and the cell pellet was resuspended in 500 μl of Milli-Q water and frozen at − 20 °C until assayed (a separate fraction was set aside for protein quantification). For the preparation of extracts, the 500-μl samples were taken to a volume of 2 ml with water, and 1 ml of 8 M NaOH and 1 ml of 25 mg/ml hydroxylamine was added. The sample was then heated at 60 °C for 30 min, and the pH was adjusted by adding 1 ml of 6 N HCl. Sequential extractions were carried out as described [44] with the exception that samples were extracted twice with 2 ml of diethyl ether and twice with 2 ml of ethyl acetate. A total of 6 μl of 5 mM undecanedioic acid was added to the collection tube to serve as an internal standard of the derivatization procedure. Once completely evaporated with nitrogen gas, the final dry residue was resuspended in 75 μl of trimethylsilyl, incubated at 60 °C for 30 min and kept at − 20 °C until injected. A total of 2-μl samples were injected into a GC-MS (Agilent Technologies ref. 7890A-5975C), with an HP-5MS 60 × 0.25 × 0.25 capillary column, using a splitless method and pressure ramp, and the results were analyzed using ChemStation GC/MSD software. The ratio between the areas was normalized by the protein concentration of the sample (μg/μl).

### Statistical Analysis

Data are expressed as mean ± SEM. Statistical significance was determined by two-way ANOVA and the Student’s *t* test, using Microsoft Excel and GraphPad Prism 6 software. A *p* value of < 0.05 was considered significant.

## Results

### Ghrelin Modulates CPT1A Activity Differentially in Cortex and Hypothalamus

**Fig. 1:**
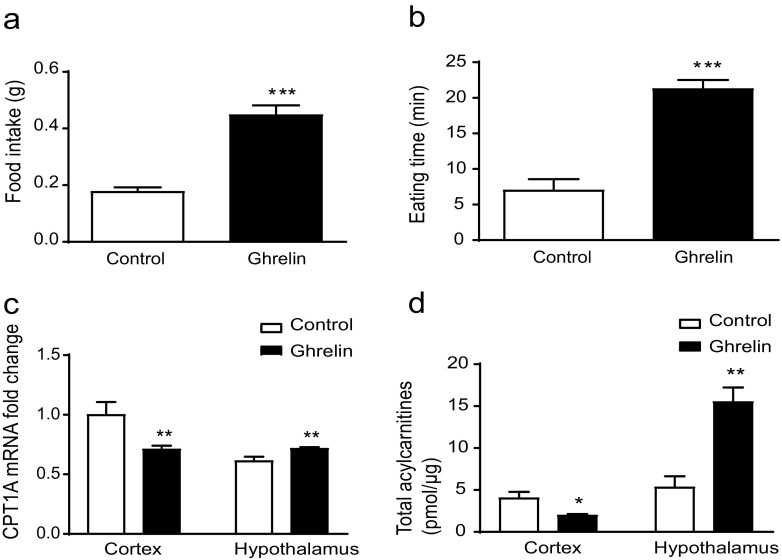
Differential effect of ghrelin in cortex and hypothalamus. **a** Changes in food intake. **b** Eating time. **c** CPT1A mRNA levels measured by qPCR (the cortex control group is the reference group). **d** Acylcarnitine levels measured by HPLC-MS/MS in mice after two ip ghrelin injections. Results are represented as mean ± SEM. *n* = 6; *p < 0.05; **p < 0.01, ***p < 0.001

To address the differential effects on CPT1A activity in the cortex and hypothalamus, we measured the CPT1A mRNA levels and total acylcarnitine content in both regions in ghrelin-treated and saline-injected control mice. As expected, ghrelin-treated mice showed a 2-fold increased food intake (Fig. 1a) and spent more time eating a chow diet (Fig. 1b). Cortical CPT1A mRNA levels were reduced up to 1.3-fold in ghrelin-treated mice and showed a slight increase in hypothalamus (Fig. 1c). Next, we measured the acylcarnitine content as an indicator of CPT1 activity in both tissues. Cortex and hypothalamus had similar acylcarnitine basal levels (4.83 ± 0.15 and 5.30 ± 0.3 pmol/μg, respectively), but in ghrelin-treated mice, cortical acylcarnitine content dropped to 48% while the hypothalamic pool increased 3-fold (Fig. 1d).

### Ghrelin Modulates GABA Shunt Enzymes Differentially in Cortex and Hypothalamus

**Fig. 2:**
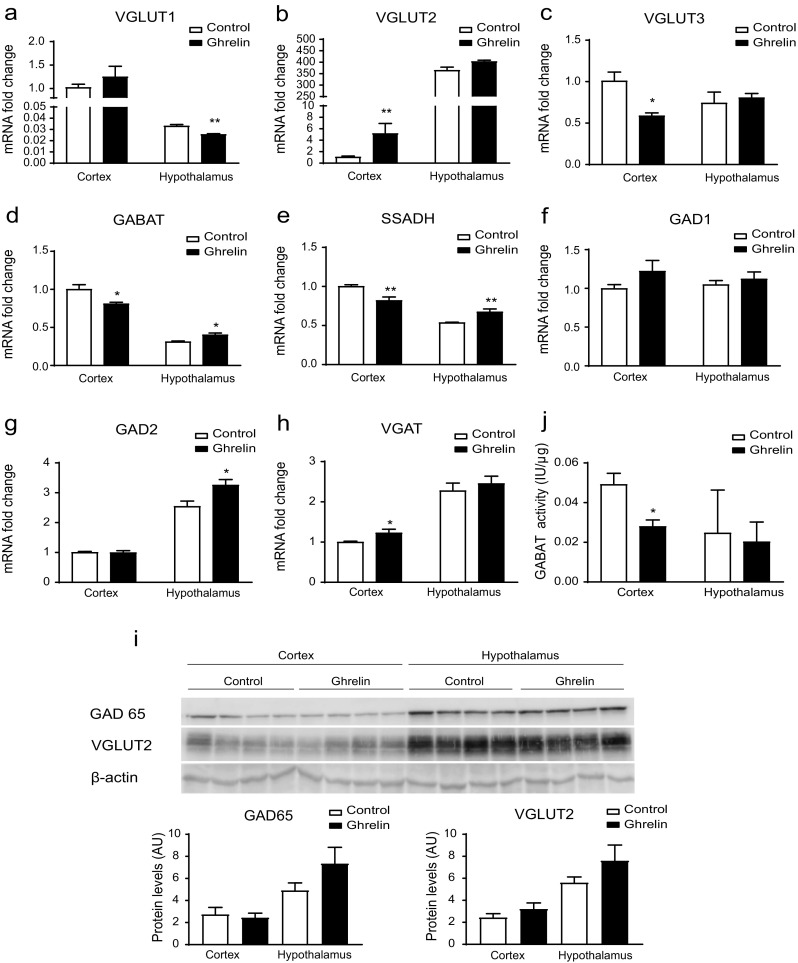
Ghrelin modifies GABA metabolism in cortex. Analysis of the following in the cortex and hypothalamus of mice after two ip ghrelin injections. **a**–**c** Relative mRNA levels of *Vglut1*, *Vglut2*, *Vglut3* genes analyzed by qPCR*.* The cortex control group is the reference group. **d**–**h** Relative mRNA levels of GABA metabolism genes. The cortex control group is the reference group *n* = 6. **j** Levels of GABAT activity *n* = 4. **i** Representative Western Blot of the protein levels of GAD65, VGLUT2, and β-actin *n* = 4. Results are represented as mean + SEM.; *p < 0.05, **p < 0.01

To determine if this change in CPT1 activity correlates with changes in the expression of genes related to glutamate and GABA, we determined the mRNA levels of different genes involved in glutamate and GABA metabolism, taking as a reference the cortex control group. Vesicular glutamate transporters (VGLUT) 1, 2, and 3 showed different patterns: VGLUT1 slightly decreased in the hypothalamus and remained unaltered in cortex (Fig. 2a); while generally expressed VGLUT2 increased 5-fold in cortex (Fig. 2b) and GABAergic neuron-associated VGLUT3 decreased up to 0.6-fold in cortex (Fig. 2c), while both remain unaltered in hypothalamus.

We also assessed the modulation of GABA metabolism by ghrelin. mRNA levels of the GABA shunt genes (GABAT and SSADH) were higher in cortex than in hypothalamus (Fig. 2d, e). In ghrelin-treated mice, cortical levels were reduced, while hypothalamic levels increased. Thus, intraperitoneal ghrelin produced differential effects in cortex and hypothalamus in terms of GABA shunt genes. However, canonical GABA generators such as GAD did not follow this pattern. GAD1 seemed to be unaffected (Fig. 2f) and GAD2 increased in the hypothalamus of ghrelin-treated mice, while it remained unaltered in cortex (Fig. 2g). Vesicular GABA transporter (VGAT) mRNA levels slightly increased in cortex, and no changes were observed in the hypothalamus (Fig. 2h). Altogether, this indicates that GABA metabolism is altered in the cortex after ghrelin’s injection.

Next, we assessed changes in protein levels of GAD65/GAD2 and VGLUT2 in the cortex in ghrelin-treated mice. Neither cortical GAD65/GAD2 nor VGLUT2 protein levels changed after ghrelin treatment (Fig. 2i). However, cortical GABAT activity drops to 58% in ghrelin-treated mice, while hypothalamic GABAT remained unaltered (Fig. 2j). These results suggest that GABA metabolism is reduced in the cortex after ghrelin’s administration.

### Ghrelin Reduces GABA Release and FAO in Primary Cortical Neurons

**Fig. 3:**
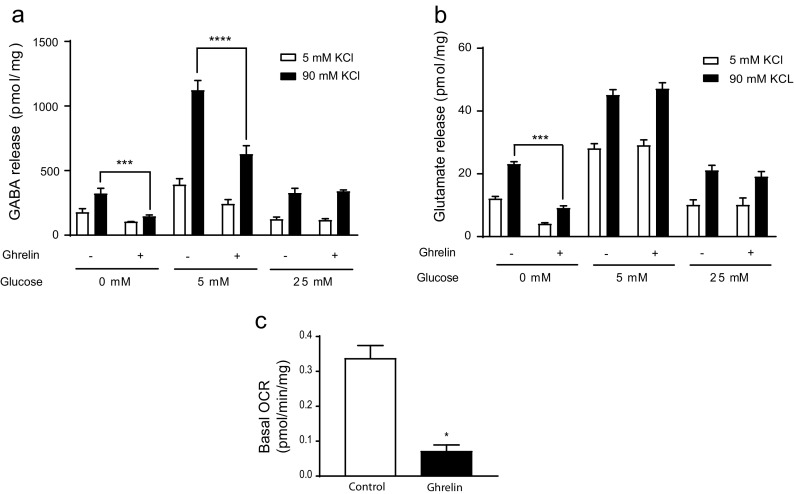
Ghrelin reduces GABA release and fatty acid oxidation in primary cortical neuronal cultures. **a** GABA release at different glucose concentrations. **b** Glutamate release at different glucose concentrations. **c** Analysis of oxygen consumption rate (OCR) after incubation with exogenous palmitate (Seahorse XF Analyser). Results are represented as mean + SEM. *n* = 4 in triplicates; *p < 0.05. ***p < 0.001

Given that the expression of GABA shunt enzymes is reduced by ghrelin in mouse cortex, we wanted to assess the direct effect of ghrelin on the GABA release of cultured primary neurons. We observed that released GABA at depolarizing 90 mM KCl concentration was significantly reduced up to 55% compared to 5 mM KCl in ghrelin-treated neurons at glycorrhachia-like glucose levels (5 mM glucose) [47] and in glucose-deprived medium (Fig. 3a). Furthermore, basal depolarized levels of released GABA were 4-fold higher at 5 mM glucose, compared to those at 0 and 25 mM glucose. Since both GABAergic and glutamatergic neurons can be found in primary cortical cultures, we assessed the effect on glutamate release as well. In the assay conditions, glutamate release was not affected at glycorrhachia-like glucose levels, but it was reduced in glucose-deprived cortical neurons (Fig. 3b). Since CPT1A expression and activity was reduced in the cortex by intraperitoneal ghrelin, we wanted to assess the effect of ghrelin on FAO. To do so, we performed metabolic extracellular flux (XF) analysis in a palmitate-, carnitine-, and glucose-enriched medium. Basal mitochondrial OCR in ghrelin-treated neurons was reduced by 80% (0.35 to 0.07 pmol/min/μg, *p* < 0.05) (Fig. 3c), which is in agreement with the reduction of CPT1 activity.

### CPT1A Ablation Mimics Ghrelin’s Effect on GABA Release in Primary Cortical Neurons

**Fig. 4:**
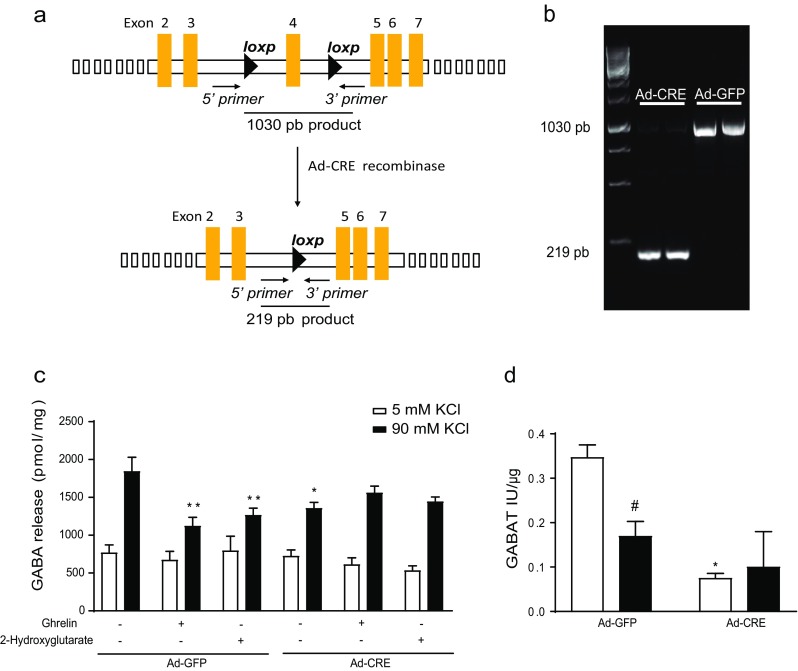
Effect of CPT1A deletion on GABA release in primary cortical neuronal cultures. Cortical neuron culture from CPT1A^(loxP/loxP)^ embryo mice were obtained and incubated with Ad-Cre-GFP or Ad-GFP (100 PFU/cell). **a** Scheme of Cre recombination product. **b** Electrophoretic analysis of the gDNA amplicon after Cre recombinase effect. **c** Levels of GABA release after ghrelin and/or 2-hydroxyglutarate incubation. **d** Levels of GABAT activity in the presence of ghrelin. *n* = 4 in triplicate. Two-way ANOVA *p < 0.05, **p < 0.01 and *t* test #*p* < 0.05

Since ghrelin treatment in cortical neurons reduced CPT1A expression, FAO, and GABA release, we wanted to assess whether the ablation of CPT1A would affect GABA release in the same direction as ghrelin. Firstly, we generated a potentially conditional CPT1A knockout mouse by taking advantage of two heterozygous stem cell clones from the cell repository of the European Conditional Mouse Mutagenesis (EUCOMM) Program. CPT1A^(loxP/loxP)^ mice were obtained from one of these clones (Supplemental Fig. 1). To assess the integrity of the *loxP* sequences, primary hepatocytes obtained from CPT1A^(loxP/loxP)^ mice were infected with CRE-expressing adenovirus (CRE). The infection successfully removed the *loxP*-flanked region containing the CPT1A exon 4, since gDNA amplicon from both sides of the homologous region dropped to 219 bp compared to control (GFP) cells 1030-bp amplicon (Fig. 4a, b). Regarding CPT1A mRNA expression, CRE-infected primary cortical neurons showed a reduction in wt CPT1A and exon4-deleted CPT1A mRNA levels (Supplemental Fig. 2d). The amplicon from exon 3 to 5 from CRE-infected cDNA with an expected 116-bp length was barely detectable after 24- and 48-h infections, which indicates great instability of the deleted CPT1A mRNA product.

Once we had generated the neuronal model, we assessed the effect of CPT1A deletion on the release of amino acid neurotransmitters from primary cortical neurons. We observed a 35% reduction in released GABA in depolarizing conditions, due to ghrelin or CPT1A deletion (Fig. 4b). Interestingly, when we blocked the tricarboxylic acid (TCA) cycle by inhibiting isocitrate dehydrogenase with 2-hydroxyglutarate at basal conditions, GABA release dropped with a similar trend to ghrelin’s effect. Analysis of mRNA levels of the GABA shunt genes did not show any change under ghrelin treatment. However, recombinase-induced CPT1A deletion significantly reduced mRNA levels of GABAT, SSADH (Supplemental Fig. 3 a, b). In addition, when we analyzed GABAT activity, CPT1A deletion reduced GABAT activity as it did ghrelin incubation (Fig. 4c). Furthermore, CPT1A deletion also reduced the mRNA levels of GAD1 involved in the canonical pathway to generate GABA out of glutamate and the mRNA levels of the GABA transporter VGAT as it did ghrelin treatment (Supplemental Fig. 3 c, e). All these results suggest that CPT1A ablation recapitulates the ghrelin’s effect on GABA metabolism and release, which indicates that CPT1A is a mediator of ghrelin’s action.

### CPT1A Deletion and Ghrelin Treatment Reduce Intracellular Citrate and α-Ketoglutarate in Primary Cortical Neurons

**Fig. 5:**
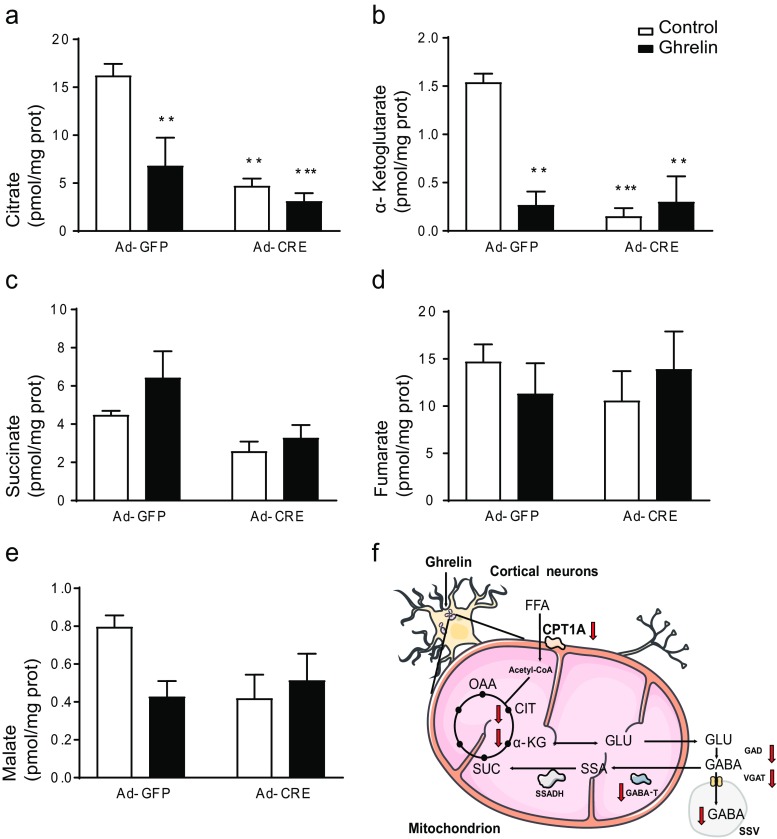
Effect of the CPT1A deletion on the Krebs cycle metabolites in primary cortical neuron culture. The levels of the components of the Krebs cycle were measured in cortical neurons from CPT1A^(loxP/loxP)^ mice incubated with Ad-Cre-GFP or Ad-GFP (100 PFU/cell) and ghrelin: **a** citrate, **b** α-cetoglutarate, **c** succinate, **d** fumarate, **e** malate. **f** Scheme of the ghrelin action in neuronal cortical cells. OAA oxaloacetate, CIT citrate, α-KG α-ketoglutarate, SUC succinate, SSA semialdehide succinate, GLU glutamate, ssv small single vesicle. Results are represented as mean ± SEM. *n* = 4 in duplicate; **p < 0.01, ***p < 0.001

Since TCA blockage produced similar effects on GABA release to those observed due to ghrelin and CPT1A deletion, we assessed the effect of both on TCA cycle intermediates. Both ghrelin and CPT1A deletion promoted a significant reduction in citrate, the main TCA cycle intermediate (Fig. 5a), and an 80% reduction of α-ketoglutarate (Fig. 5b). The other intermediates that were assessed (succinate, fumarate, and malate) remained unchanged (Fig. 5c–e).

## Discussion

The orexigenic effect of ghrelin in hypothalamus has been extensively studied [9, 48, 49]. It involves several pathways, including AMPK activation and ACC phosphorylation [50, 51], leading to reduced production of malonyl-CoA and disinhibition of CPT1A [23, 24, 27, 52–54]. This results in the production and release of orexigenic neuropeptides AgRP and NPY, and GABA neurotransmitter. Our results confirm this effect: ghrelin intraperitoneal administration induces food intake in lean mice and increases the hypothalamic acylcarnitine pool, which indicates that CPT1A is activated. Besides its orexigenic effect in hypothalamus, ghrelin may enhance motivation for food intake, since it mediates in the rewarding effect of palatable food [16–18, 55]. Systemic administration of ghrelin causes dopamine release in the nucleus accumbens, which leads to a hedonic feeling of reward that is also needed for addiction development [17, 56]. This ghrelin effect involves several brain regions like the amygdala, hippocampus, and prefrontal cortex activated by neuronal projections from the nucleus accumbens, which suggests that ghrelin has an indirect effect in these brain regions [57]. This indirect ghrelin effect could explain our results in cortical tissue where, contrary to hypothalamus, ghrelin clearly reduces CPT1A activity. However, our studies on primary culture of cortical neurons show that ghrelin has a direct effect on these neurons, reducing CPT1A and FAO. Mechanistically, GHSR1a seems to mediate such effects, as it is expressed in cortex [58]. Although not demonstrated yet in the cortex, but increasingly observed in other brain regions, the potential heterodimerization of ghrelin receptor GHSR1a with other GPCR receptors may potentially modulate specific signal transduction in discrete sets of neurons in the brain (reviewed in [59, 60]). GHSR1a heterodimerizes with at least five different GPCRs: serotonin 2C receptors attenuating orexigenic ghrelin signaling [61], dopamine D1 and D2 receptors altering dopamine signaling [62, 63] and, at peripheral level, with melanocortin 3 receptors modulating ghrelin signaling [64]. Furthermore, another important player has emerged recently: the truncated ghrelin receptor lacking transmembrane domains 6 and 7, GHSR1b. This receptor is widely expressed in many tissues where it co-localizes with the GHSR1a receptor. It has been observed that GHSR1b modulates both internalization of the active GHSR1a receptor causing the subsequent ghrelin signaling attenuation, and the ability of GHSR1a to form oligomeric complexes with other receptors, inducing changes in ghrelin-induced signaling [65]. The cellular effects of these receptor-receptor interactions remain elusive in the major regions of brain, and further investigation will show a versatile system that can transduce signals through various signaling cascades, probably depending on the cellular microenvironment that will explain the pleiotropic actions of ghrelin in different brain areas.

Cortical neurons use a wide range of neurotransmitters. We assessed the effect of ghrelin on amino acid neurotransmitters, since most cortical neurons are either GABAergic (15%) or glutamatergic (85%) [66–68]. Here, we show that ghrelin reduces GABA release at glycorrhachia-like glucose levels (5 mM) [47], while glutamate release remains unaffected. An important part of GABA production comes from TCA cycle anaplerotic pathways [69, 70]. One of the TCA anaplerotic pathways is GABA shunt. GABA shunt is highly conserved through evolution from plants to vertebrates, with varying functions among the different species [71–73]. It activates itself in pathological events such as Alzheimer’s disease [74], epileptic episodes [75, 76], and after brain ischemia [77, 78]. Physiologically, GAD activity changes have been observed during fasting in hypothalamus. Other researchers have observed changes in GABA shunt activities related to modulation of food intake: three hyperphagic rat models show increased GAD activity in VMH and two of them have increased GABA shunt activities as well [79]. These observations, together with the previous statement regarding GAD [29] and VGAT [30], indicate that GABA metabolism modulation in hypothalamus depends on the nutritional state. This modulation might be extensible to other brain areas, since caloric restriction can modulate GAD isoenzyme expression in cerebellum, superior colliculus, temporal cortex [80], and visual cortex [81]. In our study (Fig. 5f), we show that either ghrelin or a FAO reduction due to CPT1A ablation can reduce GABAergic output from cortical neurons. Ghrelin reduction of GABAergic output in cortex could explain some of the central extra-hypothalamic effects of this gastric hormone.

The functional significance of a reduction in inhibitory neurotransmitters, such as GABA, in cortical neurons under ghrelin action suggests the excitatory/inhibitory balance is adjusted within neuronal networks to function properly, and could explain animal behavior related with foraging. The anxiogenic and alertness effect needed to complement hypothalamic effects for foraging in animals [82] and to block sleep [83] are evident in a paradigm in which inhibitory neurotransmitters, such as GABA, have reduced output. In addition, the reduction in FAO and mitochondrial respiration observed in our results could be related with a neuroprotective effect associated with ghrelin and fasting [35, 84, 85]. Ghrelin’s neuroprotective effect with enhanced memory and spatial learning in mice may be closely related to mitochondrial metabolism modulation in rodents [86, 87]. At molecular level, a reduction in FAO can contribute to a reduction in the production of reactive oxygen species, protecting the cell from oxidative stress and reducing apoptosis [88, 89]. CPT1A acts as part of the mechanism by which ghrelin can modulate mitochondrial processes, since the deletion of its gene promotes deep changes in the metabolic responsiveness of the neuron to ghrelin. Consequently, CPT1A and FAO may play a role in other important ghrelin functions, such as stimulating synapsis and modulating electrical activity, which could increase cortical networks to enhance memory and cognition [41]. Further studies are needed to clarify the role of CPT1A and FAO in the molecular mechanisms involved in the various ghrelin actions.

To sum up, our data demonstrate that ghrelin produces a differential reduction in cortical GABA output, compared to hypothalamus. This reduction is produced by a drop in FAO, which produces a subsequent drop in GABA metabolism and in TCA intermediates involved in GABA production, which would explain the reduction in GABA release. This evidence suggests that the action of ghrelin on GABA is region specific within the brain, providing a basis for ghrelin differential effects in the central nervous system.

## Electronic Supplementary Material

ESM 1 (PDF 676 kb)

## Abbreviations

AMPK: AMP-dependent protein kinase
CPT: Carnitine palmitoyltransferase
FAO: Fatty acid oxidation
GABA: γ-Aminobutyric acid
GABAT: GABA transaminase
GHSR: Growth hormone secretagogue receptor
GAD: Glutamic acid decarboxylase
SSADH: Succinate semialdehyde dehydrogenase
TCA: Tricarboxylic acid
VGAT: Vesicular GABA transporter
VGLUT: Vesicular glutamate transporter
