# Supplementary material for: Ghrelin Causes a Decline in GABA Release by Reducing Fatty Acid Oxidation in Cortex
Source: Mol Neurobiol. 2018 Feb 2;55(9):7216–28. doi: 10.1007/s12035-018-0921-3 (PMC6096967; doi:10.1007/s12035-018-0921-3)
Supplement: Supplementary file 1 — (PDF 676 kb) [file 12035_2018_921_MOESM1_ESM.pdf]

Ghrelin causes a decline in GABA release by reducing fatty acid oxidation in ortex

Joan Francesc Mir[a,b], Sebastián Zagmutt[a], Mathieu P Lichtenstein[c], Judit García-Villoria[d], Minéia Weber[a], Ana Gracia[b,e], Gemma Fabriàs[f] Josefina Casas[f], Miguel López[gh], Núria Casals[i,b], Antònia Ribes[d], Cristina Suñol[c], Laura Herrero[a,b], Dolors Serra[ab*]

[a]Department of Biochemistry and Physiology, Facultat de Farmàcia i Ciències de l'Alimentació and Institut de Biomedicina de la Universitat de Barcelona (IBUB), Universitat de Barcelona, E-08028 Barcelona, Spain

[b]Centro de Investigación Biomédica en Red de Fisiopatología de la Obesidad y la Nutrición (CIBEROBN), Instituto de Salud Carlos III, E-28029 Madrid, Spain

[c]Institut d'Investigacions Biomèdiques de Barcelona, Centro de Investigación Biomédica en Red de Epidemiología y Salud Pública (CIBERESP), Barcelona, Spain

[d]Sección de Errores Congénitos del Metabolismo – IBC, Servicio de Bioquímica y Genética Molecular, Hospital Clínic, IDIBAPS, Centro de Investigación Biomédica en Red de Enfermedades Raras (CIBERER), Barcelona, Spain

[e]Nutrition and Food Science Area, School of Pharmacy, Universidad del País Vasco/Euskal Herriko Unibersitatea, Leioa, Spain

[f]Research Unit on BioActive Molecules, Department of Biomedicinal Chemistry, Institute of Advanced Chemistry of Catalonia (IQAC)/CSIC, Barcelona, Spain

[g]NeurObesity Group, Department of Physiology, CIMUS, University of Santiago de Compostela-Instituto de Investigación Sanitaria, Santiago de Compostela, 15782, Spain

[i]Departament de Ciències Bàsiques, Facultat de Medicina i Ciències de la Salut, Universitat Internacional de Catalunya (UIC), Barcelona, Spain

[*]Corresponding authors:

E-mail addresses: **dserra@ub.edu**

Dolors Serra, PhD

Department of Biochemistry and Physiology, IBUB

School of Pharmacy

Universitat de Barcelona

Av. Joan XXIII, 27-30

E-08028 Barcelona, Spain

Tel: (+34) 934 024 522

ORCID: 0000-0002-4936-4206

Supplemental Figure 1. Scheme of the obtention of CPT1A$^{(loxP/loxP)}$ mice. CPT1A$^{(+/frt\text{-}loxP)}$ mice containing the CPT1A conditional expression cassette were crossbred with C57BL6/J Flp-frt recombinase-expressing mice to eliminate the frt-*lacz* cassette and obtain CPT1A$^{(+/loxP)}$ mice. FRT: target sequences for FLP recombinase are in green; Exon Del: internal exon deletion; IRES: internal ribosome entry site; LacZ: β-galactosidase gene; pA: polyadenylation site; loxp: target sequence for CRE recombinase; hBactP: human β-actin promoter; NEO: neomycine resistance gene.

Supplemental Figure 2. ddPCR analysis of CPT1A conditional expression cassette copy number inserted into the genome of CPT1A$^{(+/frt\text{-}loxP)}$ mice. (a) Two CPT1A$^{(+/frt\text{-}loxP)}$ mice K462 and K465 and one wt mice were screened by ddPCR for the control gene *tert* and the *lacz.* (b) ddPCR quantification. (c) Electrophoretic analysis of the cDNA amplifications of the CPT1A mRNA levels in Cre-infected or GFP-infected primary cortical neurons for 24 and 48 h.

Supplemental Figure 3. Analysis of the mRNA levels of GABA metabolism genes in the primary cortical neurons with deleted CPT1A. Primary cortical neurons infected with Ad-CRE recombinase (CRE) or ad-GFP were treated with ghrelin (black bars) or without ghrelin (white bars). (a-c) Relative mRNA levels of GABA metabolism genes were analysed by qPCR. Results are represented as mean + SEM. n=4-6; * $p < 0.05$, ** $p < 0.01$ respect to the Ad-GFP control.

Supplemental Fig1

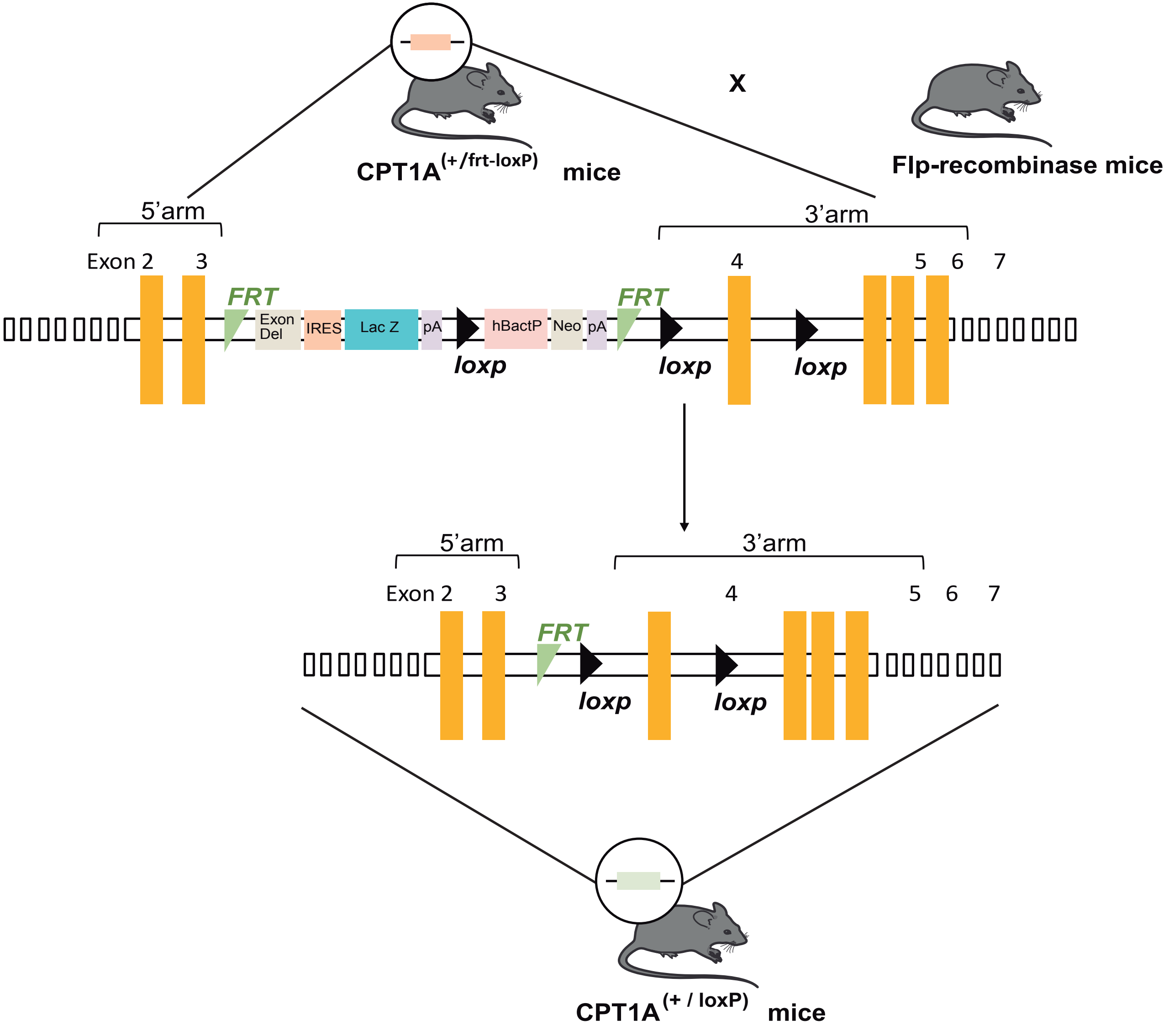

Supplemental Fig2

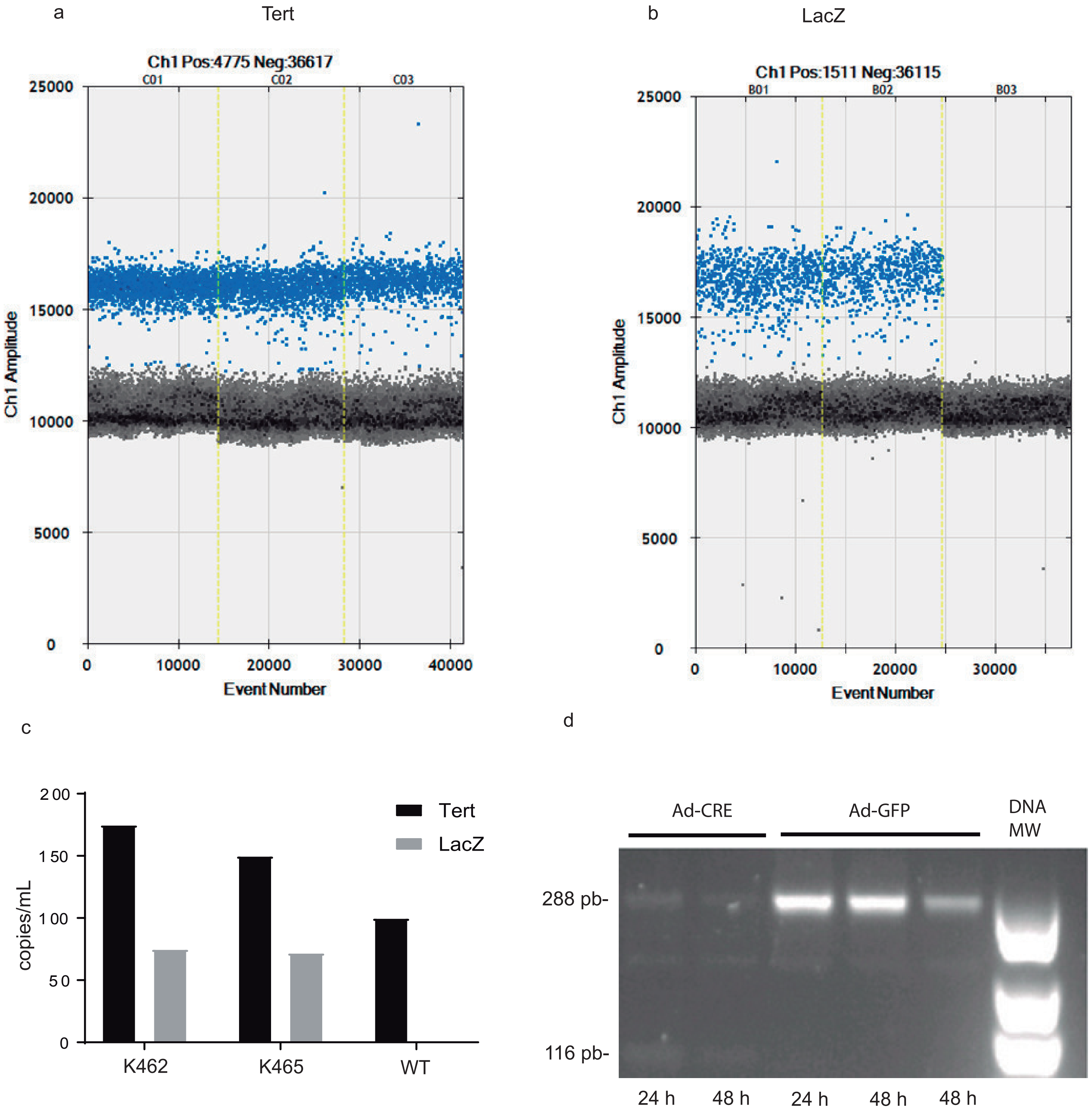

Supplemental Fig3

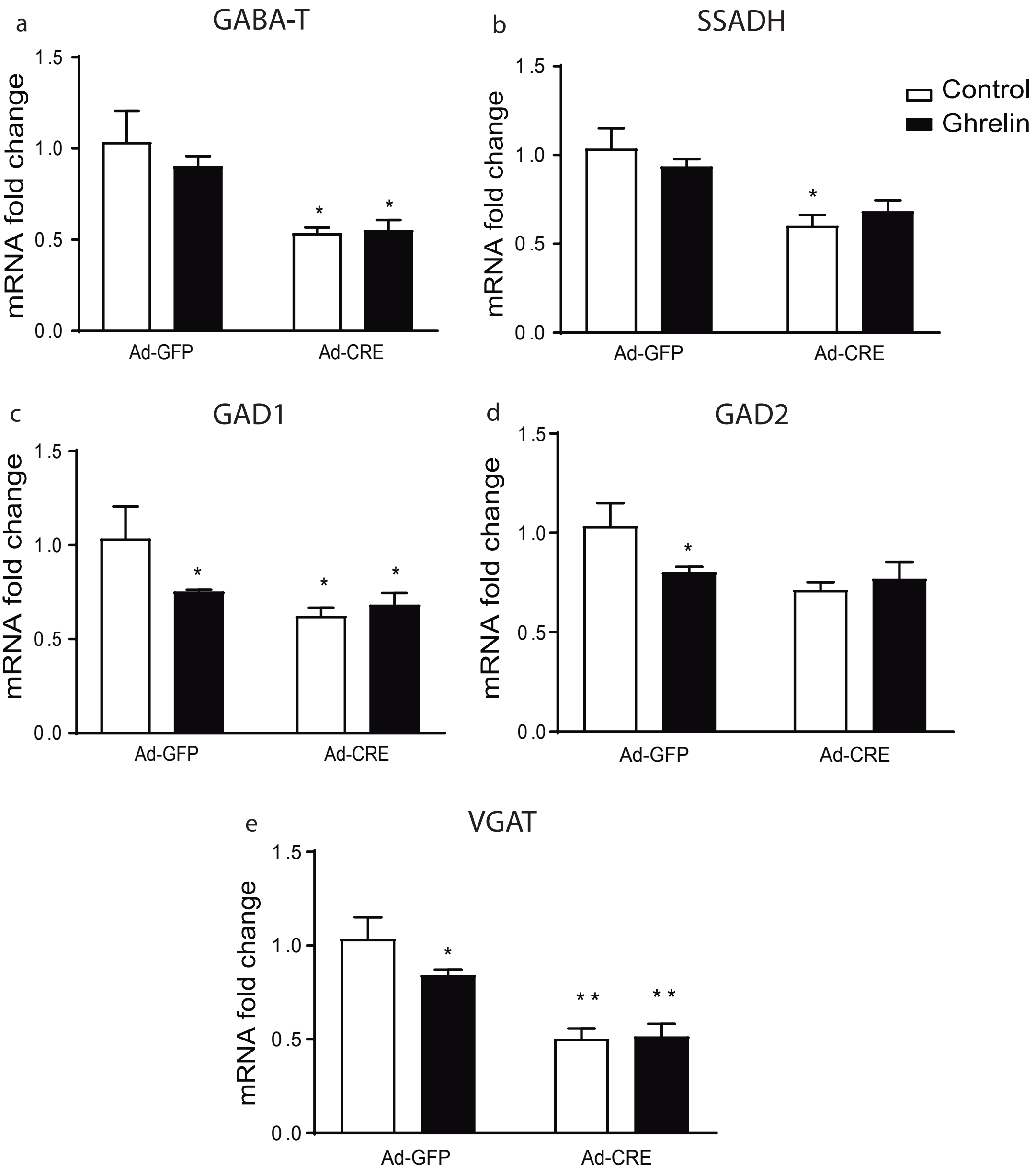

Supplemental table1:

Primers for qRT-PCR dectection and relative quantification of genes in mouse

GENE	SEQUENCE
CPT1A for	5'- GACTCCGCTCGCTCATTC
CPT1A rev	5'- AAGGCCACAGCTTGGTGA
AgRP for	5'- TGCAGACCGAGCAGAAGAAG
AgRP rev	5'- GACTCGTGCAGCCTTACACA
VGLUT1 for	5'- AAAAGCAGCAGCCAAGGTT
VGLUT1 rev	5'- CGCAGTTTAGCATTTCAGGAC
VGLUT2 for	5'- CTGGGGTCCTTGTGCAGTAT
VGLUT2 rev	5'- CCGAAGCTGCCATAGACATAG
VGLUT3 for	5'- TAAGGAGTAGCATGTTGCTCAAA
VGLUT3 rev	5'- GGCAACCACCATGTACTCTTCT
VGAT for	5'- GGCAACCACCATGTACTCTTCT
VGAT rev	5'- TGAGGAACAACCCCAGGTAG
GAD1 for	5'-ATACAACCTTTGGCTGCATGT
GAD1 rev	5'-TTCCGGGACATGAGCAGT
GAD2 for	5'-ACTAAAGAAAATGAGAGAAATCATTGG
GAD2 rev	5'-AGCATGGCATACATGTTGGA
GABAT for	5'-TGCCTCCAGAGAACTTTGTG
GABAT rev	5'-TGATGAGCTGGGACATGC
SSADH for	5'-AACAGCTGGAAAGGGGTCTC
SSADH rev	5'-CATTAAGTCGTACCATTTACGGAGT
